# Feeding Fungal-Pretreated Corn Straw Improves Health and Meat Quality of Lambs Infected with Gastrointestinal Nematodes

**DOI:** 10.3390/ani10091659

**Published:** 2020-09-16

**Authors:** Hai Xiang, Xueli Zhao, Yi Fang, Fei Wang, Rong Liang, Xuezhao Sun, Shuiping Wang, Rongzhen Zhong

**Affiliations:** 1 Jilin Provincial Key Laboratory of Grassland Farming, Northeast Institute of Geography and Agroecology, Chinese Academy of Sciences, Changchun 130102, China; xianghai@iga.ac.cn (H.X.); zhaoxueli6367@163.com (X.Z.); fangyi@iga.ac.cn (Y.F.); wangfei@iga.ac.cn (F.W.); lr13689962199@163.com (R.L.); 2 College of Advanced Agricultural Sciences, University of Chinese Academy of Sciences, Beijing 100049, China; 3 College of Animal Science, Southwest University, Chongqing 402460, China; 4 College of Animal Science and Technology, Jilin Agricultural Science and Technology University, Zuojia 132109, China; Xuezhaos@hotmail.com

**Keywords:** corn straw, gastrointestinal nematode, lamb, meat quality, white-rot fungus

## Abstract

**Simple Summary:**

Non-chemical strategies to control gastrointestinal nematode (GINs) infections are urgently needed to support the sustainable development of the livestock industry. The potential anti-parasitic properties in fungal-pretreated corn straw on health and meat quality of lambs infected with GINs were investigated in this study. In summary, feeding fungal-pretreated corn straw improved health and meat quality, including meat color and tenderness. Improved meat traits were attributed to fungal-pretreated corn straw providing additional dietary protein for lambs and secreting some nematicidal metabolites to repel GINs, which increased PCV and plasma iron content of lambs and reversed negative effects of GINs on meat quality.

**Abstract:**

Infections with gastrointestinal nematodes (GIN) adversely affect meat color in lambs. Although white-rot fungi (WRF) pretreatment increases nutritional value and fiber digestion of corn straw for lambs, whether it can improve meat quality of lambs infected with GINs is unknown. The objective of this experiment was to study effects of feeding WRF-pretreated corn straw on the health and meat quality of lambs infected with GINs. Sixteen healthy Ujumqin lambs were orally drenched with 3rd-stage GINs larvae and randomly divided into two dietary treatments of control (CON) and WRF diets for 70 days of feeding. Results showed that feeding WRF-pretreated corn straw decreased L* and b* values (*p* < 0.05) and increased a* value (*p* < 0.01) of both longissimus thoracis et lumborum (LTL) and semimembranosus (SM) muscles of lambs infected with GINs. Feeding WRF-pretreatment corn straw decreased fecal egg count (*p =* 0.014) and increased packed cell volume (*p =* 0.013) of lambs from 28 d of feeding and increased plasma iron content (*p =* 0.008) of lambs from 56 d of the feeding. Feeding WRF-pretreatment corn straw decreased myosin heavy-chain (MyHC)-I (*p =* 0.032) and MyHC-IIα (*p =* 0.025) content in LTL muscle and MyHC-I (*p =* 0.022) and MyHC-IIβ (*p =* 0.048) in SM muscle of lambs. In conclusion, although there were no significant changes in the content of most amino acids or increased intensity of better flavor compounds, meat quality and health of lambs infected with GINs was significantly improved by feeding WRF-pretreated corn straw due to increased PCV and meat color and tenderness.

## 1. Introduction

The global sheep flock reached approximately 1.36 billion individuals in 2019. The highest percentage of sheep population is in Asia (42.3%), followed by Africa (31.7%), Europe (11.0%), Oceania (8.3%) and America (6.8%). Livestock farming, especially sheep production, is the cornerstone of sustainability for rural communities in China. By 2050, agriculture was challenged to increase sheep production by over 60% to meet global food requirements [1]. Worldwide, sheep production systems are generally extensive, semi-intensive, or grazing with variable supplementation. Nearly all grazing animals on pasture-based systems are exposed to helminths. Gastrointestinal nematode (GINs) infection is a major factor limiting profitability for grazing sheep [2]. In addition to direct adverse effects on growth performance and health, GINs infection also indirectly reduces meat quality, including changed fatty acid composition and flavor of muscle, lighter meat color, increased tenderness, causing poor conformation of sheep carcasses, increased meat fattiness, decreased polyunsaturated fatty acid content and lighter meat color [3,4]. Therefore, it is necessary to decrease GIN infection in sheep to improve meat quality. Synthetic anthelmintics were widely used for decades to prevent and treat GINs. However, there are several disadvantages, including environmental pollution, resistant parasite populations, and especially chemical residues in meat. Consequently, alternative strategies are needed to control GIN infection in sheep. Many studies have focused on controlling GIN infections by means of nutritional regulation. For example, acacia (*Acacia Mill*.) extracts containing tannins were found to be effective in killing GINs in sheep [5] and it was suggested that feeding tannin-rich plants could control GIN infection [6]. Recently, biologic control of GINs in small ruminants is becoming more common, especially using nematode predatory fungi to kill nematodes [7]. *Duddingtonia flagrans* produce a large number of thick-walled chlamydospores to prevent them from being digested in sheep gastrointestinal tract, those spores could germinate into hyphae and produce a predatory trap to capture and digest nematodes [8]. In addition to *D. Flagrans*, some fungi with similar functions were explored. Nematode-trapping fungi (*Arthrobotrys oligospora*) can control nematodes [9] by absorbing urea and forming ammonia, which in turn initiates the lifestyle switch to form trap structures that capture and kill nematodes. In recent years, white-rot fungi (WRF) were found having antiparasitic activity. The potential mechanisms conclude that the mycelium of white-rot fungi (WRF), a heterogeneous group of fungi, often grew on the eggs of nematode (*Deladenus siricidicola*) in medium and prevented these eggs from hatching, significant reducing numbers of juveniles [10]. In addition, an aqueous extract of *Agaricus blazei* Murril suppressed fecal egg count of nematode in sheep [11]. However, whether the presence of WRF or their metabolites in diets can improve meat quality by reducing GINs burden in sheep remains unknown.

China is the largest agricultural country in the world with abundant biomass resources, including >800 million tons of plant residue (straw) produced annually, nearly one-third of global production. This includes up to 280 million tons of corn straw, although it has a low utilization rate due to low protein content and poor digestibility. Of the many approaches to the improvement of straw digestibility, biologic fermentation techniques seem to be the best. Compared to physical and/or chemical treatments, biologic treatment has definite advantages in terms of energy consumption and avoidance of toxic chemicals [12]. The WRF are commonly used for straw pretreatment to increase fiber digestibility, probably due to their secreted oxidative ligninolytic enzymes including laccase, manganese peroxidase and lignin peroxidase to decompose lignin [13]. We hypothesize that feeding WRF-pretreated straw can decrease GINs infection and then improve health and meat quality of sheep. Therefore, this experiment aimed to study the effects of feeding WRF-pretreated corn straw on health and meat quality of lambs infected with GINs.

## 2. Materials and Methods

### 2.1. WRF-Pretreated Corn Straw Preparation

**Figure 1:**
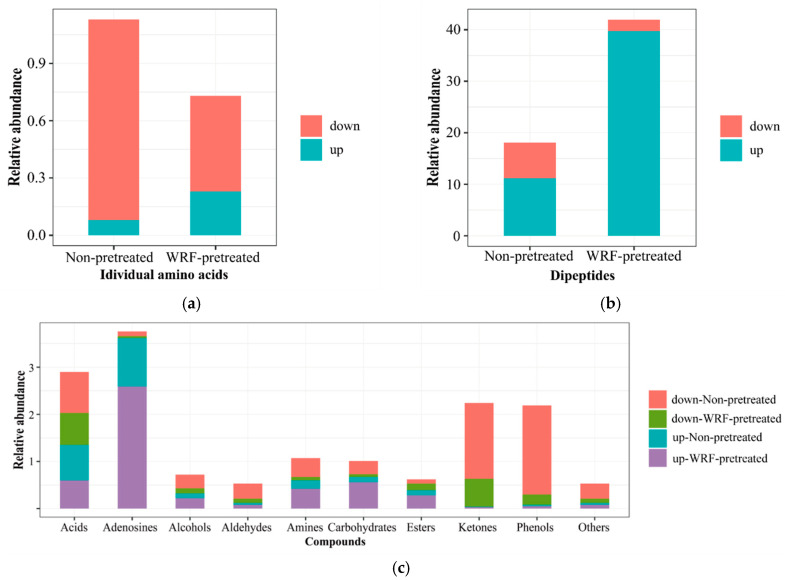
Up-or downregulated categories and relative abundance of metabolites between untreated (CON) and fungal-pretreated (WRF) corn straw. (**a**) Relative abundance of individual amino acids in CON and WRF corn straw; (**b**) relative abundance of dipeptides in CON and WRF corn straw; (**c**) Categories and the relative abundance of metabolites in CON and WRF corn straw. CON—untreated corn straw-based diet as control; WRF—replacing 40% untreated corn straw with 40% white-rot fungi-pretreated corn straw (DM basis); down—downregulated; up—upregulated.

Corn straw at late ripening stage with 89.2% dry matter (DM) content was harvested in Songyuan City, Jilin province, China and chopped into 2–3 cm lengths. Then, the substrate containing 75% corn straw, 5% soybean, 20% wheat bran, 0.5% gypsum powder, 1% lime powder and 0.5% sugar (DM basis) was prepared for WRF fermentation. The substrate was mixed with water at a ratio of 5 to 3 (weight basis) and every 550 g mixed substrate was compacted into an autoclavable bag (18 × 36 cm) by hand. All bags were autoclaved using a high-pressure steam sterilization pot (YXQ-100SII, Shanghai, China) at 121 °C for 2 h to kill bacteria and germinate the spores. Then, after cooling on a super clean bench (SW-CJ-2FD, Suzhou, China), sterile bags were stored in an air-conditioned chamber at 25°C until inoculated. The spawn of WRF (*Lentinus edodes*, strain WX1) used in the present study were purchased from Shouguang Institute of Edible Fungi (Shandong, China). Cultivation procedures were followed as described in our previous study [14]. Briefly, 8.0 g of liquid spawn was aseptically placed into each bag and mixed to distribute the spawn equally over substrate. Then, all bags were fermented at 25 °C. About 1500 bags with cultivated corn straw for the feeding experiment were pretreated for 28 days for feeding. The control corn straw was not pretreated with sterilization and cultivation. Differences in metabolite profiles (Figure 1) between control and WRF-pretreated corn straw were measured as described by Zou et al. [15] using liquid chromatography–mass spectrometry (LC-MS) (Shimadzu, LC-MS2010, Beijing, China). Briefly, 500 mg of each sample was collected within 5 min and immediately transferred into liquid nitrogen for at least 15 min of frozen and then stored at −80 °C until analysis. Six replicates were determined for each sample.

### 2.2. Lambs and Management

Animal use and experimental procedures were approved by the Animal Care Committee, Institute of Geography and Agroecology, Chinese Academy of Sciences, Changchun, China. Sixteen healthy Ujumqin lambs with a mean ± SD live weight of 31.9 ± 4.3 kg were selected and individually weighed. Prior to the formal feeding experiment, all lambs were dewormed with a combination of abamectin, albendazole and levamisole (0.2, 5.0 and 7.5 mg/kg BW, respectively) to eliminate existing GINs, with no fecal eggs subsequently identified with the McMaster technique [2]. At 28 days after deworming, all lambs were artificially orally drenched with 3rd stage larvae of mixed nematodes (0.89 ± 0.04 *Haemonchus contortus*, 0.08 ± 0.04 *Ostertagia circumcincta* and some other species of nematodes, as assessed by light microscopy) only once, which were prepared by the egg hatching procedure as described by Zhong et al. [2].

### 2.3. Experimental Design

After infection, lambs (8 lambs per diet) were given *ad libitum* access to control (CON) and WRF diets (Table 1) which was formulated according to the nutrient requirements of sheep [16]. The only difference of WRF diet from CON diet was untreated corn straw replaced with WRF-treated corn straw. The entire experiment consisted of two periods: 7 d of adaption and 70 d of formal feeding and sample collection period. All lambs were fed individual in the signal pen (2.0 m long and 2.0 m width), except for metabolic experiment and feed were provided twice daily at 06:00 and 18:00. During metabolic experiment, each lamb was confined in a single metabolic cage (140 cm long, 100 cm wide and 124 cm high) with ad libitum access to fresh water and feed. At each meal, the concentrate was offered first and then sufficient untreated corn straw or WRF-pretreated corn straw was provided to ensure that each lamb would have no more than ~10% refusal. In the next morning before feeding, all orts including concentrate and straw were weighed and recorded. Chemical analyses of diets were conducted using the methods of the Association of Official Analytical Chemists [17] for dry matter (DM), ash, ether extract and crude protein (CP). Neutral detergent fiber (NDF) and acid detergent fiber (ADF) contents were determined according to Van Soest et al. [18].

### 2.4. Sampling Procedures

During the experiment, before the morning feeding on Days 1, 7, 14, 28, 42, 56 and 70, blood samples (5 mL) were collected from the jugular vein of each lamb by venipuncture into heparin-containing vacutainers for hematological measurements, these tubes were immediately centrifuged at 2500× *g* at 4 °C for 10 min. Plasma was separated and stored at −20 °C until further analysis. In addition, 2-mL blood samples were also collected into anticoagulant vacutainers with EDTA-K2 and packed cell volume (PCV) was measured. After blood collection, fecal sample (10 g) was collected directly from the rectum of each lamb and determined fecal egg count (FEC).

After 70 d of feeding, all lambs were fasted for 12 h and then were electrically stunned and slaughtered by exsanguination under commercial conditions. The electric stunning was applied using a head-only stunner with the stunning electrodes between the eyes and ears on either side of the head. The current was delivered at a constant voltage of 220 V, 1.0 A for 3 s with scissor tongs. Lambs were exsanguinated within 20 s after stunning and then were hung to remove their skin, head (at the atlanto-occipital joint), forefeet (at the carpal–metacarpal joint) and hind feet (at the tarsal–metatarsal joint). The left side of carcass was used for measurements of meat quality. Samples of 300 g of left longissimus thoracis et lumborum (LTL) muscle and 300 g of left semimembranosus (SM) muscle were collected. Approximately 50 g of each sample was kept in the dark at 4 °C for measuring pH at 24 h post-mortem, shear force and meat color, ~10 g of each meat sample was immediately frozen in liquid N2 for at least 3 h and then moved to −80 °C for protein expression related to different myosin heavy-chain (MyHC) and the remaining meat of each sample was cut into 3 parallel samples and stored at–20 °C for the chemical composition of intermuscular fat (IMF), volatile flavor compounds and amino acids (AA) composition.

### 2.5. Analytical Procedures

Plasma iron contents were measured using a multiparameter automated hematology analyzer (7020, Hitachi, Beijing, China) with commercial kits (Meikang Biotechnology Co., Ltd., Ningbo, China). The FEC was determined using the McMaster’s technique as described by Zhong et al. [19] with results expressed as eggs per gram (EPG) of feces. The PCV of whole blood was determined using a UNICO-hematocrit centrifuge (182-E, Dayton, NJ, UAS) and a microcapillary reader (Damon/IEC Division).

The pH values of the LTL and SM muscles were tested post-mortem at 24 h (pH_24_) using a pH meter (PHS-3C, Jiangsu Jiangfen Electroanalytical Instrument Co., Ltd., Jiangyan, China), calibrated with pH 4.6 and 7.0 buffers. Each muscle sample was tested 3 times at different locations. The LTL and SM muscle samples were cut into strips (~1.27 cm diameter and 20 mm long) and sheared parallel to the muscle fiber direction. At 24 h post-mortem, shear force was measured using a muscle tenderness meter (XU155, Xieli Science and Technology Co., Ltd., Qinhuangdao, China). Each sample of LTL and SM, muscles were tested 3 times and values averaged. For each muscle, meat color traits of L* (lightness), a* (redness) and b* (yellowness) were measured with a CR410 chroma meter (Konica Minolta Sensing, Inc., Japan) calibrated with a standard white plate before measuring. Three replicates of each sample at different points were measured and the average value was used.

Intermuscular fat (IMF) was determined by extracting the sample with petroleum ether using an automatic soxhlet extractor (SER148, VELP, Beijing, China) after being freeze dried for 24 h at -50 °C with a vacuum freezing dryer (ScanVac CoolSafeTM, LaboGene, Ltd., Lynge, Denmark). This Soxhlet extraction was operated as described by Hopkins et al. [20]. Briefly, ~0.3 g freeze-dried meat sample was extracted in 80 mL petroleum ether for 4 h and then the residue was heated in an oven at 105 °C for 30 min to remove residual petroleum ether.

The AA profiles of meat were analyzed using an automatic amino acid analyzer (Hitachi L-8900, Beijing, China), as described by Jiao et al. [21]. Briefly, 0.1 g of dry meat sample was placed in a hydrolysis tube containing 10 mL of hydrochloric acid (6 M), 4 drops of phenol solution added, frozen at −20 °C for 3 min, evacuated for 5 min, nitrogen added, hydrolyzed for 22 h and then cooled to room temperature. Hydrolysate was filtered in a constant volume of 50 mL and 1.0 mL of the filtrate dried in vacuo to dissolve 1.0 mL of ammonium citrate buffer and pass through a 0.22-μm filter membrane. Ion exchange chromatography column (855–3294, Hitachi, Beijing, China), where the amino acids were eluted by the sodium buffer system. After reaction with ninhydrin, the synthesized derivatives were simultaneously measured at wavelengths of 440 and 570 nm. Due to the low response at 570 nm, the proline derivative was detected at 440 nm.

The volatile flavor compounds in the meat sample were measured using Headspace solid phase micro-extraction (SPME) coupled with gas chromatography-mass spectrometry (GC–MS) as described by Guo et al. [22] with minor modifications by our lab. Briefly, SPME with 65 µm PDMS/DVB fiber (Supelco, Bellefonte, PA, USA) was used to extract volatiles from 2-g samples at 70 °C for 30 min. After absorption, the fiber was stuck to a GCMS-QP2010 gas chromatograph mass spectrometer (Shimadzu, Tokyo, Japan) at 250 °C for 4 min during desorption. The carrier gas was helium and the flow rate was 1.4 mL/min. HP-5MS column (0.25 mm internal diameter, 30 m length, 0.25 µm film thickness, Shimadzu), oven temperature was operated for 5 min at 40 °C, increased to 230 °C at 3 °C/min for 5 min, was used to separate volatile compounds with a total acquisition program of 73 min. The electron impact energy was set at 70 eV at 10 microscan/s and data were collected in the 33–300 m/z range. Concentrations of various volatile flavor compounds were quantified according to volatile peaks.

The MyHC content (MyHC-I, IIα, IIβ and IIx) in each meat sample was measured by ELISA (Rayto, RT-6100, Jinan, China) with commercial kits (Shanghai Enzyme Biotechnology Co., Ltd., Shanghai, China), as described by Depreux et al. [23] with appropriate modifications. Briefly, after melting, 0.5 g of sample was added to PBS buffer (1-mM Na2HPO4, 1.6-mM NaH2PO4, 0.15-M NaCl, pH 7.4), the sample was thoroughly homogenized and centrifuged for 15–20 min at 3000× *g* and 4 °C to collect the supernatant. Protein content of MyHC extractions was determined according to the manufacturer’s instructions. Then, setting standard wells with 50 μL of various concentration standard liquids and test sample wells with 50 μL of supernatant sample (sample diluted 5 times with diluent) separately (sample or horseradish peroxidase was not added in blank well). In addition to the blank wells, 100 μL of detection antibody labeled with horseradish peroxidase was added to each of the standard and sample wells. Reaction wells were covered with sealing film and incubated at 37 °C in a water bath for 60 min. After each well was thoroughly washed with washing solution, chromogenic reagent A and B were added to each well to incubate for 15 min at 37 °C in the dark. Finally, 50 μL of stop solution was added to each well and within 15 min, OD value of each well was measured at 450 nm.

### 2.6. Statistical Analyses

Data of meat parameters were analyzed using a General Linear Model of SAS [24]. Data of blood parameters of each lamb were analyzed using the mixed model procedure described by Littell et al. [25] with a model consisting of treatment, sampling time and treatment × tine interaction as fixed effects and animal as the random effect. Figures were drawn using R 3.5.3 and Excel 2010. Measurements of blood parameters obtained from each lamb at different sampling times were treated as repeated measurements. The EPG data were log-transformed (ln (EPG + 1)) prior to statistical analyses. Means were separated using the least squares mean and differences were declared for *p* ≤ 0.05.

## 3. Results

### 3.1. Fecal Egg Count, Packed Cell Volume and Plasma Iron Content of Lambs

**Figure 2:**
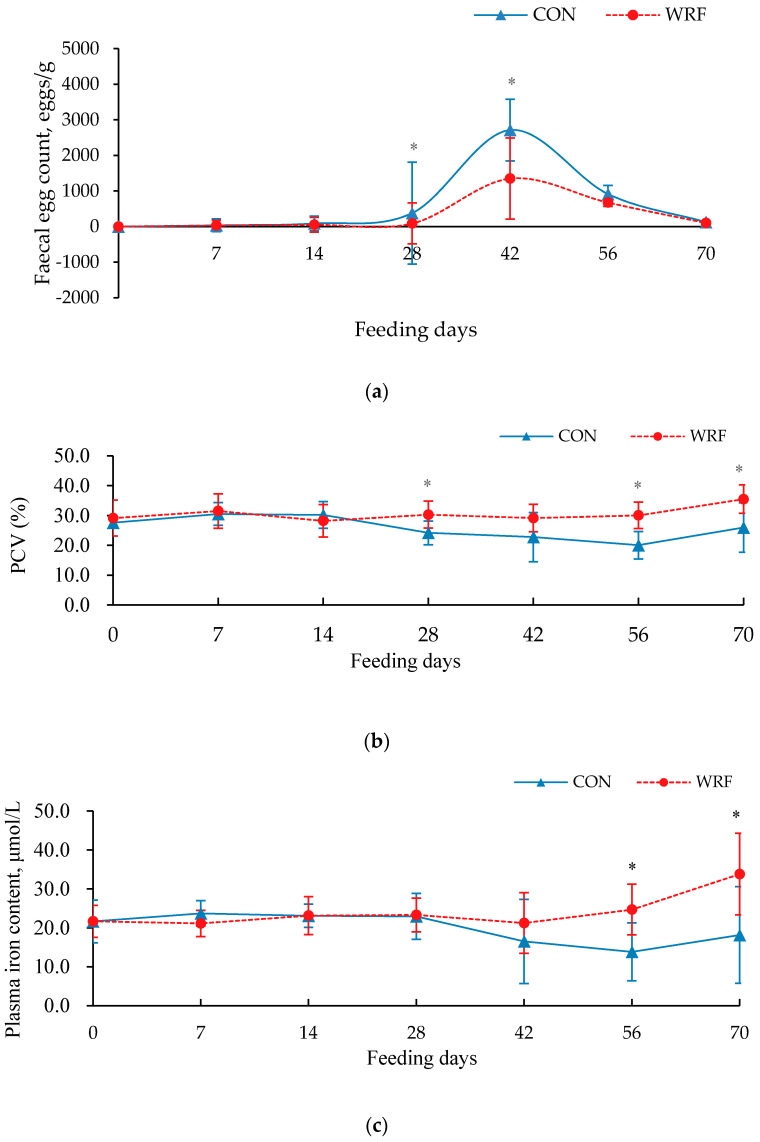
Mean ± SEM effects of dietary-fungal-pretreated corn straw on (**a**) fecal egg count (FEC), (**b**) packed cell volume (PCV, lower panel) and (**c**) plasma iron content (upper panel) of lambs infected with gastrointestinal nematodes; difference between treatments (* *p <* 0.05).

From Day 7 to 70, FECs in WRF-pretreated lambs were numerical lower than those of CON lambs Figure 2a and the effects of feeding WRF-pretreated on FECs at Day 28 and 42 were significant (*p* < 0.05). From Days 1 to 42, there were no differences between treatments in plasma iron content (*p* > 0.05) (Figure 2c). However, plasma iron content in WRF lambs was increased on Days 56 (*p* = 0.008) and 70 (*p* = 0.016) of the feeding period than those of CON lambs. The PCV of lambs in both groups did not change in the first 2 weeks of feeding (*p* > 0.05) Figure 2b, but PCV of WFR lambs increased at Days 28 (*p* = 0.013), 56 (*p* < 0.002) and 70 (*p* = 0.007) of the feeding period.

### 3.2. Traits of pH Shear Force, Intermuscular Fat and Color of Lamb Muscles

Feeding WRF-pretreated corn straw affected meat color and shear force of LTL and SM muscles (Table 2). Regarding meat color, L* and b* values of both muscles of WRF lambs were lower (*p* = 0.014 for L* and *p* = 0.001 for b* in LTL muscles, *p* value = 0.008 for L* and *p* = 0.002 for b* in SM muscles) than those of CON lambs. Feeding WRF-pretreated corn straw increased a* value of both muscles (*p* < 0.001 in LTL muscles and *p* = 0.007 in SM muscles) compared to CON lambs and it also increased shear force of LTL (*p* < 0.001) and SM muscles (*p* = 0.001). However, for both muscles, there were no differences between groups for pH_24_ or IMF.

### 3.3. Amino Acid Profiles in Muscles of Lambs

Although non-essential amino acids (NEAA) and total amino acids (TAA) contents in WRF lambs were slightly higher than those of CON lambs (Table 3), almost all individual AA content was unaffected, except for cysteine, which was higher (*p =* 0.020) in WRF lambs.

### 3.4. Myosin Heavy-Chain Content in Muscles of Lambs

**Figure 3:**
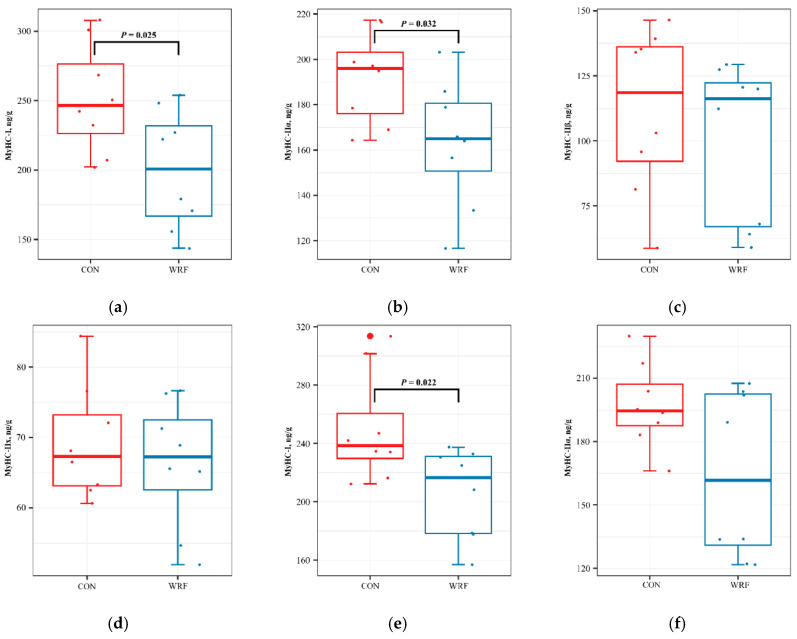

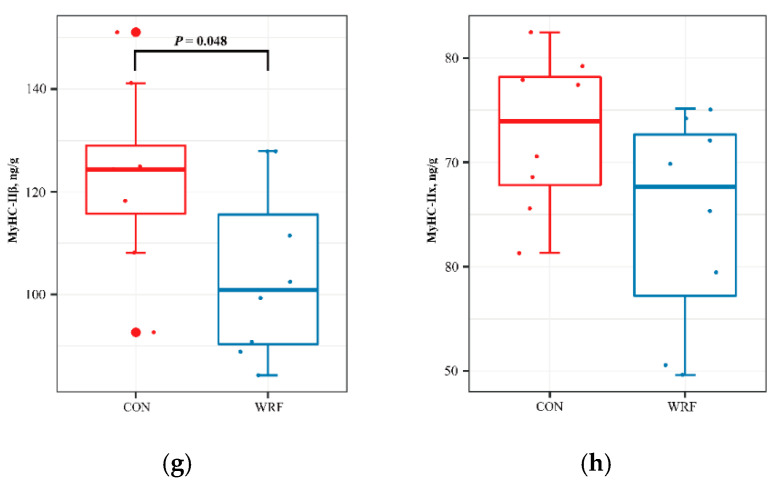
Effects of dietary fungal-treated corn straw on myosin heavy-chain (MyHC) content in longissimus thoracis et lumborum (LTL) and semimembranosus (SM) muscles of lambs infected with gastrointestinal nematodes. (**a**) MyHC-I content in LTL muscle of CON and WRF lambs; (**b**) MyHC-IIα content in LTL muscle of CON and WRF lambs; (**c**) MyHC-IIβ content in LTL muscle of CON and WRF lambs; (**d**) MyHC-IIx content in LTL muscle of CON and WRF lambs; (**e**) MyHC-I content in SM muscle of CON and WRF lambs; (**f**) MyHC-IIα content in SM muscle of CON and WRF lambs; (**g**) MyHC-IIβ content in SM muscle of CON and WRF lambs; (**h**) MyHC-IIx content in SM muscle of CON and WRF lambs; Within a box-plot, *p* value was given to declare significance which *p <* 0.05 means differ.

Feeding WRF-pretreated corn straw decreased MyHC-I, MyHC-IIα, MyHC-IIβ and MyHC-IIx contents in fresh LTL muscle (*p* = 0.025, 0.031, 0.470, 0.502, respectively) and SM muscle (*p* = 0.022, 0.053, 0.048, 0.085, respectively) (Figure 3).

### 3.5. Volatile Compounds in Muscles of Lambs

**Figure 4:**
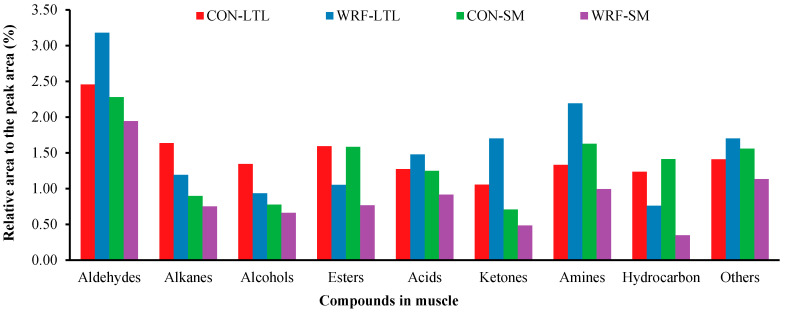
Effects of dietary-fungal-pretreated corn straw on volatile compounds in fresh longissimus thoracis et lumborum (LTL) muscle and semimembranosus (SM) muscle of lambs infected with gastrointestinal nematodes.

A total of 126 volatile compounds were quantified in both groups. There were 20 mutual volatile compounds in two muscles and two groups, plus additional 40 and 31 volatile compounds that uniquely existed in WRF and CON muscles, respectively. All quantified volatile compounds were divided into the following categories: aldehydes, alkanes, alcohols, esters, ketones, amines, acids, hydrocarbon and other volatile compounds (Figure 4). Effects of feeding WRF-pretreated corn straw on proportion changes of various kinds of compounds were different in the two muscles. Overall, feeding WRF-pretreated corn straw increased proportions of aldehydes, acids, ketones and amines in LTL muscle, but decreased their proportions in SM muscle when compared to the CON group. However, the change trend of alkanes, alcohols, esters and hydrocarbon were consistent in both muscles.

## 4. Discussion

We recently reported that the nutritive value and in vitro rumen lignin digestibility of WRF-pretreated corn straw was significantly higher than untreated corn straw. In particular, CP content in WRF-pretreated corn straw increased by 40.5–68.4% and the in vitro lignin digestibility increased 40% depending on various fungi when compared with untreated corn straw [14]. In the present study, differences in metabolites between WRF-pretreated and untreated corn straw were characterized. In total, 392 metabolites were identified in Control and WRF-pretreated corn straw and these metabolites were categorized into 12 classes, including 112 dipeptides, 76 acids, 25 amines, 24 individual amino acid, 18 esters, 16 ketones, 15 alcohols, 13 adenosines, eight carbohydrates, five phenols, three aldehydes and other metabolites (Figure 1). Among all these identified metabolites, 245 metabolites were upregulated and 147 metabolites were downregulated in WRF-pretreated versus Control corn straw. The biggest difference was that more upregulated and less downregulated individual amino acids and dipeptides were detected in WRF-pretreated versus Control corn straw. In addition, esters, alcohols, ketones, amines, adenosines and carbohydrates in WRF-pretreated corn straw were upregulated more than those of Control corn straw, whereas, all downregulated categories in WRF-pretreated corn straw were less than those of un-pretreated corn straw. Based on changes in metabolite profile, nutritive value, especially AA and peptides of corn straw were optimized by WRF pretreatment.

In our previous study, we reported that GIN infections decreased a* values and total heme pigment content of lamb meat due to some blood-sucking nematodes such as *Haemonchus contortus* will result in anemia and blood iron loss; therefore, GIN infections had negative effects on meat color. Meanwhile, many studies have found that feeding plant-derived-polyphenols could alleviate such negative effects on meat by eliminating GIN infections [19,26]. Consequently, in the present study, we hypothesized that reducing GIN burden by changing the diet would improve meat color. Some studies have attempted to improve meat quality of GIN-infected sheep by increasing dietary protein to mount a strong immunity associates to GINs. For example, feeding bananas without any additional protein resulted in infected lamb’s meat with lower b* and L* value and higher a* value compared to dietary protein supplementation [27]. Furthermore, supplemental dietary protein decreased L* values and increased a* values of lamb meat [3]. Overall, dietary protein supplementation can improve meat quality of lambs infected with GINs [28]. Consistent with our hypothesis, in the present study, WRF lambs had significantly lower L* and higher a* values in the two tested muscles. There are two proposed causes. First, feeding WRF-pretreated corn straw reduced the numbers of parasitic GINs, based on lower FEC and higher PCV of WRF lambs. Various fungi, including *Ascomycota*, *Zygomycota*, *Basidiomycota* and *Chytridiomycota*, were antagonistic to nematodes and their eggs, using them as nutrition sources [10]. In addition, some chemicals have antagonistic relationships toward nematodes. For example, 2-decenedioic acid in *Pleurotus ostreatus* (a wood-rotting Basidiomycete) was isolated as a nematicidal compound. In addition, two fatty acids, namely linoleic acid and S-coriolic acid were the nematicidal compounds formed by *Pleurotus pulmonarius* [29]. Based on the present metabolite profiles, WFR-pretreated corn straw may contain these nematicidal compounds to control GINs. Second, infection with GINs may result in a total net loss of about 12 g protein/day, which consist of a net loss of about six grams form the gastrointestinal tract and a further loss of about six grams through increased AA oxidation in the body [30]. However, a WRF-pretreated corn straw-based diet can provide more dietary protein, thereby counteracting protein utilization by GINs [14]. When WRF are in a nitrogen-limited environment, communal GINs may provide essential nitrogen for communal fungi [31]. As mentioned above, to a certain extent, WRF can offset the damage caused by GINs to protein supply and demand for sheep and further improve meat color, although further research is needed to provide more insights.

Plasma iron content is closely related to total muscle iron content and can be used as an indicator of changes in meat color. Heme iron in myoglobin is directly proportional to redness of meat [32]. When heme increases, heme iron in myoglobin also increases, which may finally increase a* and decrease L* under extreme anemia conditions [33]. Therefore, actors causing plasma iron deficiency alter meat iron content and meat color. The blood sucking nematode *H. contortus* is of primary concern, as it can cause anemia. Each *H. contortus* can suck ~0.05 mL blood from its host sheep daily [34], causing iron deficiency anemia and hypoproteinemia. Lambs infected with *H. contortus* had lower serum iron content than uninfected lambs [35], similar to the previous results, decreases in L* and b* and increases in a* in the present may be due to increased total iron content of muscles. Perhaps WRF-pretreated corn straw alleviated GIN-induced anemia in lambs and thus improved meat quality. In addition, iron is also a cofactor of catalase in the antioxidant system and has an important role in preventing oxidation of lipids and maintaining meat quality [36].

In the present study, the pH_24_ of meat from both dietary treatments were all within the acceptable range (5.6–6.4) for lambs [37], implying pH was not affected by consumption of WRF-pretreated corn straw. Similarly, WRF did not affect muscle pH at 24 h in beef cattle [38]. Deposition of IMF is usually slow and mainly affected by nutrition. In the present study, IMF was not significantly affected, perhaps due to the relatively short feeding period (70 d). Tenderness is the most important to consumer’s perceptions of acceptability. Compared to pork or chicken, tenderness is even more important for red meats such as beef and lamb, as red meat contains many muscle fibers and much connective tissue. We previously reported that *H. contortus* infection decreased shear force of lamb from 27.65 N (un-infected lambs) to 18.88 N, with meat softer than the normal. In meat from 6-month-old Ujumqin lambs, shear force ranged from 30.2 to 35.7 N [26,39]. In the present study, WRF-pretreated corn straw improved tenderness of lamb meat, indicating that feeding WRF-pretreated corn straw is beneficial to improve meat quality.

Meat quality should generally have an integrated evaluation, as most parameters are closely related and affected by many physiological processes, especially in ruminants. Distribution and conversion of muscle fiber types is related to meat tenderness. Four distinct single MyHC isoforms were identified, including slow-oxidative MyHC-I, fast-oxidative MyHC-IIα, fast-oxidative glycolytic MyHC-IIx and fast-glycolytic MyHC-IIβ. Increased content of MyHC-I and MyHC-IIα in muscle fibers increased meat tenderness, whereas MyHC-IIβ had the opposite effect [40]. Furthermore, muscle fiber transition from MyHC-IIβ to MyHC-I may cause more tenderness [41]. In the current study, total expression of all four kinds of MyHC was lower in WRF than CON and the proportion of MyHC-I and MyHC-IIα slightly decreased, whereas the proportion of MyHC-IIβ and MyHC-IIx was greater in the two test muscles from sheep consuming WRF versus CON. The MyHC data partially explained changes in shear force. Meat color is mainly determined by pigments, including myoglobin and hemoglobin. Contents of these two pigments are high in oxidized fiber. When the proportion of oxidized fibers in muscle increase, the muscle color is bright red and the meat color score is higher. On the contrary, the content of MyHC-IIβ fiber myoglobin and hemoglobin is low. When the proportion of MyHC-IIβ content in muscle increase, the muscle color appears pale and the meat color score is low [42]. However, in the present study, meat color was not significantly affected by MyHC proportions and feeding WRF-pretreated corn straw eliminated deleterious effects of GINs on meat color.

In addition to color, tenderness and pH, flavor is a critical component of meat acceptability. Volatile flavor compounds primarily determine the aroma and thus flavor attributes of cooked meat. Meat flavors are thermally derived and the flavor of cooked meat is attributed to the combined sensation of two very important reaction products: Maillard reaction and thermal degradation of lipids. The Maillard reaction is a series of reactions between free amino groups and reducing sugars in meat. More than half of the volatile aroma compounds in cooked meat are produced through thermal degradation of lipids [43]. Meat flavor is a combination of aroma and tastes. Aroma strongly promotes the acceptance of lamb, with a specific mutton aroma related to the presence of volatile compounds that are affected by diet [44]. Aldehydes, ketones and carbohydrates are produced from lipid oxidation. Aldehydes are usually the main source of volatile components that define the aroma of ruminant meat, with various aldehydes having distinct aromas [45]. Ketones and carbohydrates are often the main contributors to increased rancidity in meat [46]. Esters usually have creamy, sweet and appealing flavor [45]. Phenols and acids can contribute to pastoral flavor and produce an unpleasant aroma [47]. In the present results, although the proportion of aldehydes in WRF meat was largely increased, it was still difficult to conclude whether feeding WRF-pretreated corn straw improved meat flavor, because for SM muscle, all kinds of volatile flavor compounds were lower in WRF than CON, whereas for LTL muscle, aldehydes, acids, ketones and amines were higher in WRF than CON. Contrasting effects on different muscles may be due to different physiological processes, including differences in lipid and protein metabolites and impact of diets on muscle varying with muscle type [48]. Data regarding effects of WRF on meat flavor are limited and further studies are needed.

Amino acids not only contribute to the nutritional value of meat but also significantly contribute to its flavor and taste. It is well established that meat quality is affected by dietary amino acids content and their profile. Cysteine content in meat is closely related to its flavor generation [49]. Sulfur-containing compounds, e.g., cysteine, are important contributors to meat flavor because their degradation products have very low odor threshold values and therefore extremely low amounts can substantially change meat aroma [43]. In addition, cysteine is closely related to physiological responses of ruminants infected with GINs. The GIN infections cause strong immune responses in hosts, increasing host protein requirements, especially for sulfur-containing amino acids such as cysteine [29]. Thus, cysteine may be an essential nutrient in lambs infected with GINs. Miller et al. [50] suggested that cysteine can increase peripheral eosinophilia and abomasal globular leukocyte count, both of which can reduce parasite burdens. Although the AA profile in meat is rarely affected by diets, we found that dietary WRF-pretreated corn straw had higher cysteine content in LTL muscle than CON. Perhaps dietary WRF metabolites mitigated negative influences of GINs on the hosts, enabling the host to transfer more cysteine from the immune process to the growth process.

## 5. Conclusions

In conclusion, feeding WRF-pretreated corn straw improved health and meat quality of lambs infected with GINs, including increased PCV and plasma iron content and improved meat color and tenderness, but did not significantly enhance favorable flavor compounds in meat. Improved meat traits were attributed to WRF-pretreated corn straw providing additional dietary protein for lambs and secreting some nematicidal metabolites to repel GINs, which increased PCV and plasma iron content of lambs and ultimately eliminated negative effects of GINs on meat quality.

## Figures and Tables

**Table 1:** Ingredients and chemical composition of the experiment’s diets.

Feed Ingredients (% DM Basis)	CON Diet	WRF Diet
Ingredients (%)		
Corn straw	40.0	0.0
Fungal-pretreated corn straw	0	40.0
Corn meal	21.0	21.0
Soybean meal	10.2	10.2
Corn germ meal	9.0	9.0
Sunflower seed meal	5.0	5.0
Wheat bran	10.5	10.5
Limestone	1.0	1.0
CaHPO_4_	0.8	0.8
NaCl	0.5	0.5
Minerals and vitamins salt	2.0	2.0
Chemical composition (n = 8)		
Dry matter (% of the feed)	97.8	97.3
Crude protein (% of dry matter)	9.3	12.8
Ether extract (% of dry matter)	5.4	5.2
Neutral detergent fiber (% of dry matter)	51.7	48.7
Acid detergent fiber (% of dry matter)	24.1	23.9
Starch (% of dry matter)	22.6	20.8
Metabolizable energy (MJ/kg of dry matter)	13.5	14.8

CON diet—untreated corn straw-based diet as control; WRF diet—replacing 40% untreated corn straw with 40% white-rot fungi-pretreated corn straw (DM basis); Mineral salt and vitamins—purchased from Agriportal, were comprised of (per kg): 16.5 g Ca, 8.5 g P, 11.5 g Na, 1.6 g Mg, 1.5 g K, 1.7 g S, 1.25 g Fe, 1.22 g Mn, 1.23 g Z, 240 mg Co, 1750 mg Cu, 450 mg I, 50 mg Se, 350,000 IU/Ib vitamin A, 55,000 IU/Ib vitamin D3 and 500 IU/Ib vitamin E.

**Table 2:** Effects of dietary-fungal-pretreated corn straw on traits of longissimus thoracis et lumborum (LTL) and semimembranosus (SM) muscles of lambs infected with gastrointestinal nematodes.

Item	Treatments		SEM	*p* Value
	CON	WRF		
LTL muscle				
pH_24_	5.75	5.78	0.142	0.883
L*	31.6 ^a^	26.8 ^b^	1.195	0.014
a*	12.7 ^b^	18.2 ^a^	0.483	<0.001
b*	4.55 ^a^	3.07 ^b^	0.265	0.001
Shear force (N)	21.7 ^b^	30.5 ^a^	1.140	<0.001
IMF (%)	3.10	3.03	0.378	0.892
SM muscle				
pH_24_	5.77	5.84	0.084	0.557
L*	30.3 ^a^	23.2 ^b^	1.632	0.008
a*	15.7 ^b^	18.4 ^a^	0.595	0.007
b*	4.73 ^a^	3.04 ^b^	0.304	0.002
Shear force (N)	27.5 ^b^	37.3 ^a^	1.370	0.001
IMF (%)	3.10	2.55	0.316	0.236

^a,b^ values within a row with different superscripts differ significantly *(p <* 0.05); CON—untreated corn straw-based diet as control; WRF—replacing 40% untreated corn straw with 40% white-rot fungi-pretreated corn straw (DM basis); SEM—standard error of means.

**Table 3:** Effects of dietary-fungal-pretreated corn straw on amino acid profiles in longissimus thoracis et lumborum (LTL) muscle of lambs infected with gastrointestinal nematodes.

AA Profile (% of Dry Meat)	Treatments		SEM	*p* Value
	CON	WRF		
Lys	5.75	5.66	0.147	0.673
Met	0.90	0.68	0.091	0.100
Cys	0.59 ^b^	0.74 ^a^	0.040	0.020
Phe	1.99	2.02	0.050	0.660
Thr	5.10	5.16	0.122	0.723
Val	3.10	2.97	0.100	0.358
His	1.83	2.26	0.184	0.122
Arg	3.85	4.04	0.129	0.311
Leu	5.08	4.95	0.117	0.432
Ile	2.91	2.84	0.069	0.466
EAA	31.49	32.57	0.634	0.818
Asp	3.42	3.56	0.092	0.312
Ser	2.67	2.78	0.082	0.373
Glu	9.68	9.97	0.239	0.419
Gly	3.17	3.01	0.139	0.434
Ala	4.22	4.09	0.107	0.430
Tyr	2.86	2.91	0.108	0.767
Pro	2.72	1.00	0.105	0.086
NEAA	28.74	29.32	0.767	0.611
TAA	60.24	61.89	1.343	0.689

^a,b^ values within a row with different superscripts differ significantly *(p <* 0.05); CON—untreated corn straw-based diet as control; WRF—replacing 40% untreated corn straw with 40% white-rot fungi-pretreated corn straw (DM basis); SEM—standard error of means; EAA—essential and semi-essential amino acids; NEAA—non-essential amino acids; TAA—total amino acids.
